# Application of High-Z Gold Nanoparticles in Targeted Cancer Radiotherapy—Pharmacokinetic Modeling, Monte Carlo Simulation and Radiobiological Effect Modeling

**DOI:** 10.3390/cancers13215370

**Published:** 2021-10-26

**Authors:** Wei Bo Li, Stefan Stangl, Alexander Klapproth, Maxim Shevtsov, Alicia Hernandez, Melanie A. Kimm, Jan Schuemann, Rui Qiu, Bernhard Michalke, Mario A. Bernal, Junli Li, Kerstin Hürkamp, Yibao Zhang, Gabriele Multhoff

**Affiliations:** 1 Institute of Radiation Medicine, Helmholtz Zentrum München-German Research Center for Environmental Health (GmbH), 85764 Neuherberg, Germany; alexander.klapproth@helmholtz-muenchen.de (A.K.); kerstin.huerkamp@helmholtz-muenchen.de (K.H.); 2 Center for Translational Cancer Research, Technische Universität München (TranslaTUM), Klinikum Rechts der Isar, Einsteinstr. 25, 81675 Munich, Germany; stefan.stangl@tum.de (S.S.); maxim.shevtsov@tum.de (M.S.); alicia.hernandez-schnelzer@tum.de (A.H.); 3 Department of Radiation Oncology, Technishe Universität München (TUM), Klinikum Rechts der Isar, Ismaningerstr. 22, 81675 Munich, Germany; 4 Personalized Medicine Centre, Almazov National Medical Research Centre, 2 Akkuratova Str., 197341 Saint Petersburg, Russia; 5 Laboratory of Biomedical Nanotechnologies, Institute of Cytology of the Russian Academy of Sciences (RAS), Tikhoretsky Ave., 4, 194064 Saint Petersburg, Russia; 6 Department of Diagnostic and Interventional Radiology, Technische Universität München (TUM), Klinikum Rechts der Isar, 81675 Munich, Germany; melanie.kimm@med.uni-muenchen.de; 7 Department of Radiology, University Hospital, Ludwig-Maximilians-Universität München, 81337 Munich, Germany; lijunli@mail.tsinghua.edu.cn; 8 Physics Division, Department of Radiation Oncology, Massachusetts General Hospital (MGH) & Harvard Medical School, Boston, MA 02114, USA; jschuemann@mgh.harvard.edu; 9 Department of Engineering Physics, Tsinghua University, Beijing 100084, China; qiurui@tsinghua.edu.cn; 10 Research Unit Analytical BioGeoChemistry, Helmholz Zentrum München-German Research Center for Environmental Health (GmbH), 85764 Neuherberg, Germany; bernhard.michalke@helmholtz-muenchen.de; 11 Gleb Wataghin Institute of Physics, State University of Campinas, Campinas 13083-859, SP, Brazil; mbernalrod@gmail.com; 12 Key Laboratory of Carcinogenesis and Translational Research (Ministry of Education), Department of Radiation Oncology, Peking University Cancer Hospital & Institute, Beijing 100142, China; zhangyibao@pku.edu.cn

**Keywords:** cancers, gold nanoparticles, X-rays, cmHsp70.1, conjugation, targeted radiotherapy, enhancement effect, pharmacokinetic model, Monte Carlo simulation, radiobiological modeling

## Abstract

**Simple Summary:**

High-Z gold nanoparticles show potential as radiosensitizers in the radiotherapy of cancer. In this paper, we introduce the benefits and procedures for the application of gold nanoparticles in targeted cancer radiotherapy. Based on microscopic images of the distribution of antibody-conjugated nanoparticles, we established pharmacokinetic models simulating the biodistribution of nanoparticle conjugates in the tumor and tumor environment in preclinical models. This information has been implemented in radiation transport Monte Carlo simulation codes for further investigating physical and chemical enhancement and radiobiological effects, such as DNA strand breaks and cell survival. Future perspectives and challenges of translating this promising gold nanoparticle-aided radiotherapy into clinical practice are also discussed.

**Abstract:**

High-Z gold nanoparticles (AuNPs) conjugated to a targeting antibody can help to improve tumor control in radiotherapy while simultaneously minimizing radiotoxicity to adjacent healthy tissue. This paper summarizes the main findings of a joint research program which applied AuNP-conjugates in preclinical modeling of radiotherapy at the Klinikum rechts der Isar, Technical University of Munich and Helmholtz Zentrum München. A pharmacokinetic model of superparamagnetic iron oxide nanoparticles was developed in preparation for a model simulating the uptake and distribution of AuNPs in mice. Multi-scale Monte Carlo simulations were performed on a single AuNP and multiple AuNPs in tumor cells at cellular and molecular levels to determine enhancements in the radiation dose and generation of chemical radicals in close proximity to AuNPs. A biologically based mathematical model was developed to predict the biological response of AuNPs in radiation enhancement. Although simulations of a single AuNP demonstrated a clear dose enhancement, simulations relating to the generation of chemical radicals and the induction of DNA strand breaks induced by multiple AuNPs showed only a minor dose enhancement. The differences in the simulated enhancements at molecular and cellular levels indicate that further investigations are necessary to better understand the impact of the physical, chemical, and biological parameters in preclinical experimental settings prior to a translation of these AuNPs models into targeted cancer radiotherapy.

## 1. Introduction

The exceptionally rapid development of the innovative techniques in radiology and radiotherapy which have followed Wilhelm Conrad Röntgen’s discovery of X-rays at the end of 19th century has enabled a considerably deeper understanding of human diseases and improved the treatment and management of patients with cancer [1]. Radiotherapy remains one of the most common and effective treatment modalities for cancer. It has been used for both conventional treatments which target and kill malignant cells, and for improving diagnosis using contrast agents that are based on materials having a high atomic number (high-Z) [2,3,4,5]. As the dose delivered by ionizing irradiation interacting with high-Z material can be amplified via an enhancement of the photoelectric effect, the release of so-called Auger electrons and X-ray fluorescence induced electrons can increase the lethal effect of irradiation at lower doses. Gold, with an atomic number of 79, is the most studied material in this respect, as its high-Z value makes AuNPs prime candidates as contrast agents for computed tomography (CT) [6,7,8,9]. Regulla et al. [10,11] tested the effect of a gold foil and the experimental as well as simulation data showed a dose enhancement factor of up to 150 in the first tissue layer close to the gold foil. In 2004, Hainfeld et al. [12] applied AuNPs as an agent for a selective amplification of the radiation dose in tumors and were the first to demonstrate an enhanced radiosensitization effect in tumors of mice that had been irradiated with kilovoltage (kV) X-rays. In 2005, Cho [13] proposed a theoretical Monte Carlo (MC) simulation which focuses on the radiation dose deposited by secondary and Auger electrons around AuNPs after the interaction of gold with X-rays. Since then, AuNPs have become very popular for studying radiation sensitization not only due to the strongly enhanced physical dose and beneficial biological effects, but also due to the presumed biocompatibility and the production of chemical radicals induced by secondary electrons [14]. This concept is termed as “gold nanoparticle-assisted radiation therapy” or “gold nanoparticle-aided radiotherapy” [15,16,17].

Theoretical simulations using different MC codes have since been carried out by many researchers, with the results demonstrating a physical dose enhancement around AuNPs after irradiation with various radiation types, such as kV and MV X-rays, protons and heavy ions, and radionuclides [13,18,19,20,21,22,23,24,25,26,27]. After the irradiation of a single AuNP in liquid water with a very narrow beam, MC simulation studies have shown a dose enhancement factor (DEF; also denoted as dose enhancement ratio, DER), of up to 64 at a distance of 1 nm [28] and up to 500 at a distance of 10 nm [29] from the surface of the AuNP. For multiple AuNPs in liquid water or in the microenvironment of a cell, the DEF ranged between 20 and 75 within a 1 μm water shell in the proximity of the surface of the AuNPs [25,28,29]. This elevated radiation dose has the capacity to damage cell membranes, induce DNA damage and consequently result in tumor cell death [30]. Apart from the initial physical tracks, solvated electrons and other chemical species are produced that can diffuse to distant locations and attack biological sensitive moieties such as DNA [31,32,33,34]. To understand and effectively exploit the effects of a targeted uptake of AuNPs into tumor cells in order to improve radiosensitization in clinical applications, a roadmap which documents the state of art and future research directions has been outlined [35]. More recently, an updated roadmap and summary on the high-Z metal nanoparticles in clinical radiation therapy has been published [28,36].

Multiple experimental studies of AuNPs in different cell lines (in vitro*)* and mice (in vivo*)* using different radiation beams have reported an enhanced biological efficacy [17,37,38,39,40,41,42,43,44,45,46,47]. In general, irradiation induced effects, such as DNA damage and apoptosis, are enhanced by the application of AuNPs, and the overall survival of tumor-bearing mice has been shown to be improved. In addition to kV X-rays, other radiation types, such as megavoltage X-rays, protons, and heavy ions, have also been applied in combination with AuNPs [48,49,50]. However, a number of factors, such as the shape, size, precise location of AuNPs, and their biodistribution in different cell types, are known to affect the dose enhancement effect, and as a consequence the DEF has to be determined for each individual experimental setup individually [35,38,48,51]. Furthermore, potential toxic side effects of different types of nanoparticles have to be considered before they can be applied in clinical practice [52]. However, the translation of AuNPs as radiosensitizers into clinical cancer radiotherapy is limited by the lack of tumor-specific targets which enable the selective uptake of AuNPs into tumor cells.

In this regard, the Multhoff laboratory at the Klinikum rechts der Isar, Technical University of Munich (TUM) has previously reported that the major stress-inducible heat shock protein 70 (Hsp70) is selectively expressed on the membrane of a large range of different tumor cells, but not on cells in the surrounding healthy tissues [53,54]. Furthermore, standard treatment procedures, such as radiotherapy and chemotherapy, selectively further increase the expression density of membrane Hsp70 on tumor cells, but not on normal cells [55]. Moreover, an elevated Hsp70 membrane density has also been detected on relapsed tumors, metastases, and highly aggressive tumors such as triple negative breast cancer cells [56]. The unique monoclonal antibody (mAb) cmHsp70.1 is able to detect membrane-bound Hsp70 on viable tumor cells and recent work has indicated that following binding, cmHsp70.1 antibody conjugated AuNPs are rapidly taken up into membrane Hsp70-positive tumor cells by endocytosis [57]. Furthermore, stressed glioma cells exhibiting an increased expression of membrane Hsp70 preferentially take up superparamagnetic iron oxide nanoparticles (SPIONs) that are coupled to the cmHsp70.1 antibody [58]. Membrane expressed Hsp70 therefore provides an ideal tumor-specific antigen for targeting cmHsp70.1 mAb coupled AuNPs into triple negative breast cancer cells.

Targeted and non-targeted AuNPs have been used as an X-ray contrast agent for molecular computed tomography (CT) imaging of cancer [59,60,61], for X-ray fluorescence computed tomography (XFCT) [61,62,63,64,65] of tumors in mice, as well as for cone-beam XFCT simulations and experimental studies [66,67,68]. A high-sensitivity benchtop X-ray fluorescence imaging platform for detecting AuNPs inside tumors has been developed and the feasibility of L-shell XRF-based quantitative imaging of AuNPs at concentrations as low as 0.007  mg/cm^3^ (7 ppm) in biological tissues and liquid water demonstrated [64]. Recently, cmHsp70.1 mAb conjugated AuNPs have been used in a spectral CT system to image accumulated AuNPs in mouse tumors [69].

Herein, we summarize the outcome of studies carried out at the Klinikum rechts der Isar, TUM and Helmholtz Zentrum München in cooperation with other institutes and universities and discuss work in progress. These studies are based on the conjugation of AuNPs with the cmHsp70.1 mAb with the aim of targeting cancer cells to improve tumor imaging [57]. Second, we present a pharmacokinetic model for SPION nanoparticles in mice. Parallel to the experimental investigations, we present MC simulation results of the electron spectra, energy deposition, dose enhancement of single AuNPs in liquid water after being irradiated with X-rays, as well as simulated doses and biological effects of AuNPs distributed in cells. Finally, we present a multi-scale methodology to estimate the enhanced biological effects of AuNPs in a xenograft mouse phantom.

## 2. Materials and Methods

In this section, the experimental methods and technologies used for conjugation and imaging are outlined. We present multi-scale MC simulation methods for dose enhancement and mathematical modeling for pharmacokinetics of nanoparticles in the body of mice and cell survival fraction.

### 2.1. Collection of Biological Datasets

The preparation of cmHsp70.1 mAb-functionalized AuNPs and their biological properties in vitro and in vivo has been previously published [57,69], but is briefly summarized in the following sections.

#### 2.1.1. Conjugation of AuNPs with cmHsp70.1 mAb

To direct the nanoparticles towards the tumor in vivo, AuNPs were functionalized by utilizing the cmHsp70.1 mAb (multimmune GmbH, Munich, Germany) which selectively binds to membrane Hsp70 on tumor cells [57]. Briefly, spherical gold nanoparticles were coated with polyethylenglycol (PEG)-amine (Nanopartz, Loveland, CO, USA), activated using maleimide (Pierce, Thermo Fisher Scientific, Rockford, IL, USA), and incubated with sulfhydryl-activated antibodies. A PEG8 spacer separated the nanoparticles from the active binding side of the cmHsp70.1 mAb. The size distribution and quality of the mAb-coupled AuNPs were analyzed by dynamic light scattering (DLS, Zetasizer NanoS, Malvern Instruments, Malvern, UK).

#### 2.1.2. Analysis of AuNP Uptake

Murine GL261 glioblastoma, 4T1 mammary carcinoma and CT26 colon carcinoma cells were cultured in RPMI-1640 medium supplemented with 10% (*v/v*) fetal calf serum (FCS), 2 mM L-glutamine, 1 mM sodium pyruvate, and antibiotics (100 ug/mL streptomycin and 100 IU/mL penicillin) at 37 °C (5% (*v/v*) CO_2_, 95% humidity). Cells were placed on pre-coated poly-L-lysine glass slides and incubated with cmHsp70.1 mAb-functionalized AuNPs, isotype control IgG1-functionalized AuNPs or non-coated AuNPs (at Au concentration 25 μg/mL) for 1, 3, 6, 12, and 24 h at 37 °C. Following incubation, cells were washed with PBS and fixed in 0.5% (*w*/*v*) paraformaldehyde (PFA). Nuclei were additionally stained with Hoechst 33342 (Thermo Fisher Scientific, USA) or DAPI. Cells were mounted in the fluorescent mounting medium (Dako North America Inc., Carpinteria, CA, USA) and analyzed using a Leica TCS SP8 confocal microscope (Leica Microsystems, Heidelberg, Germany) and transmission electron microscopy (TEM).

### 2.2. Pharmacokinetic Models

#### 2.2.1. Model for SPIONs

SPIONs have multiple applications in cancer therapy and diagnostics [70,71,72,73,74]. We developed a compartmental pharmacokinetic (PK) model to investigate the biodistribution of SPIONs after two different injection modes, intratumoral and intravenous [75]. For this, we initially implanted GL261 glioblastoma cells into the right flank of female C57BL/6 mice. When tumors had reached a volume of 50 mm^3^, ^99m^Tc-ferucarbotran SPIONs were injected intravenously and ^89^Zr-Perimag^®^-COOH SPIONs were injected intratumorally or intravenously in different experiments. For each set of experiments, data were collected for several organs in order to determine the number of SPIONs that reached an organ at a certain time point. The data from these experiments were used to develop the PK model, which is a mathematical model based on first-order ordinary differential equations describing the number of SPIONs in organs over a period of time. The model parameters were computed using parameter fitting of the experimental data. The model was used to simulate computation data and validate the model’s accuracy by comparing simulated and experimental data. Because the hydrodynamic diameter, shape, surface charge, and Z-potential of the SPIONs and AuNPs were comparable, the images gained in TEM and spectral-CT can be used to set up the PK model also for AuNPs.

#### 2.2.2. Proposed Compartmental Model for Biodistribution of Targeted AuNPs in Cells

The data used for the structure of the compartmental model of the intracellular distribution of the nanoparticles have been generated by quantitative analysis of light microscopic images, as described previously [57].

**Figure 1:**
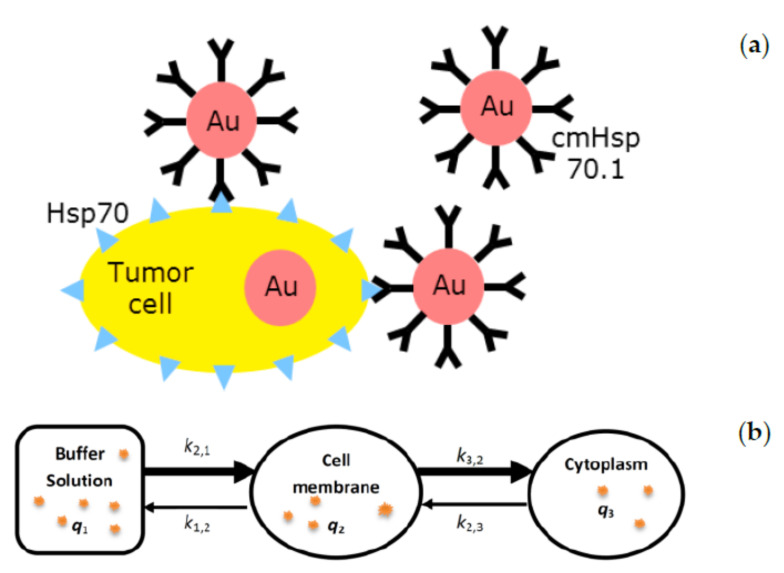
Developing a compartmental model (**b**) for analysing the mechanism of uptake of AuNPs in a cancer cell after integrating the experimental data (**a**).

According to the mechanism of the cmHsp70.1 mAb-AuNP-conjugate interaction with tumor cells (Figure 1a), we propose a cellular compartmental model to describe measured concentrations of AuNPs in the tumor cell membrane and the cytoplasm, as shown in Figure 1b. The transfer rates (*k*) can be estimated by fitting the model to the in vitro data.

### 2.3. Gold Nanoparticle Based Spectral Computed Tomographic Imaging

The tumor cell-binding cmHsp70.1 mAb-AuNPs, control IgG1-AuNPs, and unconjugated (blanc) AuNPs were injected into mice bearing CT26 subcutaneous (sc) tumors. Mice were scanned using spectral computed tomography [69]. Briefly, AuNP mediated images were acquired by a laboratory-based Philips spectral-CT scanner. The technical establishment of the spectral-CT analysis is described in detail by Schlomka et al. [76]. In short, the axial scans over 360° at a beam voltage of 100 kVp were acquired from a preclinical spectral photon-counting CT system (Philips Healthcare, Hamburg, Germany). A threshold was set at the k-edge energy of gold to optimally discriminate the signals of AuNPs. Osirix^®^ MD v10.0.5 software (Pixmeo SARL, Bern, Switzerland) was utilized for image processing. A normal background signal was measured from various regions of interest (ROIs) in the spleen. Consequently, the AuNP amounts (μg gold/mm^3^) were assessed over the total volumes of the tumors.

### 2.4. Monte Carlo Simulation of Enhanced Radiosensitization Effect

#### 2.4.1. Spectra of X-rays Used in Simulations

**Figure 2:**
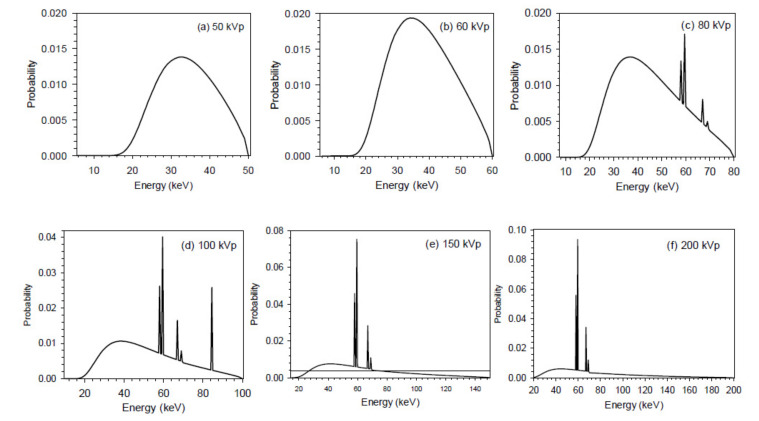
X-ray spectra generated at (**a**) 50 kVp, (**b**) 60 kVp, (**c**) 80 kVp, (**d**) 100 kVp, (**e**) 150 kVp and (**f**) 200 kVp using the SpekCalc program [73]. The energy bin width in the energy x-axes in all spectra is 500 eV.

In this work, we applied the SpekCalc program [77] to simulate an X-ray tube with a tungsten target. In Figure 2, the generated X-ray spectra of 50 kVp, 60 kVp, 80 kVp, 100 kVp, 150 kVp, and 200 kVp were presented. These spectra were implemented into the MC codes as radiation sources. The parameters utilized for generating the X-ray spectra are presented in Table 1. The default values of 0.68 and 0.33 were used for the model parameters ‘Nf’ and ‘P’ in the GUI interface [78].

#### 2.4.2. Dose Enhancement by a Single AuNP

**Figure 3:**
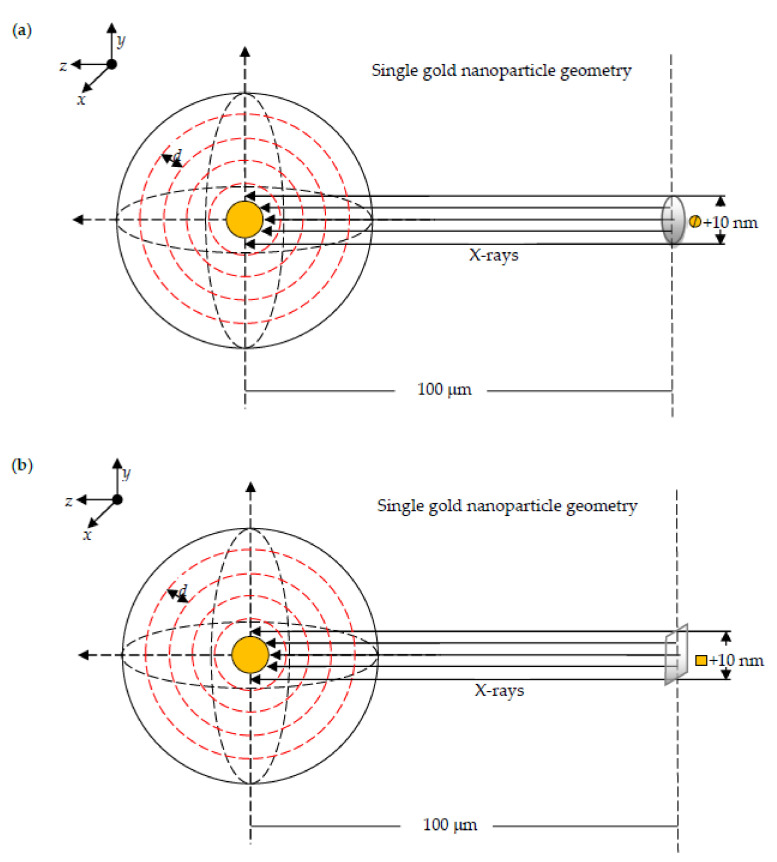
Geometric setup in MC simulation for a single AuNP irradiated with X-ray beams. The single AuNP with a diameter of 50 nm and 100 nm is placed in a liquid water phantom. X-ray beams are sampled from (**a**) a circular planar source and (**b**) a square planar source. The diameter and the perimeter of the planer source are equal to the AuNP diameter plus 10 nm. The sampled beams irradiate the AuNP along the *z*-axis in a right-handed Cartesian reference system. The distance between the center of the circular and square planar X-ray sources and the center of the single AuNP is defined as 100 μm. Energy deposited in the concentric spherical shells of thickness *d* (10 nm and 1 mm) around the AuNP is scored.

The geometry setup used in the simulation is depicted in Figure 3. One single spherical geometry filled with gold was used as an AuNP and this was located in the center of a liquid water volume. The AuNP was supposed as being pure gold, without coating and not conjugated with other molecules or antibodies. This simple assumption of a pure AuNP facilitates the comparison of simulated results, as it avoids such effects of the energy absorption in the coating molecules and the antibodies. Two particle sizes having diameters of 50 nm and 100 nm were simulated.

A plane-parallel X-ray beam irradiates a spherical AuNP. The central axis of the beam was aligned to the center of the AuNP. The energy peaks of the X-ray beams were 50 kV and 100 kV. The beams were sampled from a circular plane (Figure 3a) and a square plane (Figure 3b) source positioned 100 µm from the AuNP center. The source diameter of the circular plane and the source perimeter of the square plane were set equal to the diameter of the AuNP plus 10 nm. These definitions of the sources should increase the interaction probability of the X-rays with the AuNP. In the same geometrical conditions without an AuNP, simulations of the interaction of liquid water and X-rays were performed separately.

Energetic electrons escaping from the gold particle were scored as electron spectra around a single AuNP. To estimate the energy deposition and dose in the nanometer and micrometer ranges around a single AuNP, we defined 100 and 50 concentric spherical 10 nm-thick and 1 μm-thick shells respectively in liquid water around the surface of the single AuNP. Based on these energy depositions in the water shell for both irradiation methods with and without AuNP, it was possible to calculate the dose enhancement ratio.

In this work, three MC codes, namely PENELOPE-2018 [79], Geant4-DNA [80], and NASIC [81], were used to calculate the aforementioned quantities. PENELOPE is a condensed history simulation code, and Geant4-DNA and NASIC track are structure simulation codes. The cuff-off energy was set as 50 eV, 10 eV, and 10 eV for electrons and 50 eV, 10 eV, and 10 eV for photons in the simulation codes, PENELOPE, Geant4-DNA, and NASIC, respectively. The cross section used in PENELOPE and Geant4-DNA is from the Penelope library and NAISC uses its own electron cross sections in liquid water, and photons from the Geant4-DNA library. For a more detailed physical list and options used in the simulation, reference can be made to the comparison exercise work performed by multiple MC codes within a EURADOS framework [29].

#### 2.4.3. Method for Dose and DNA Strand Breaks for Multiple AuNPs in Cells

**Figure 4:**
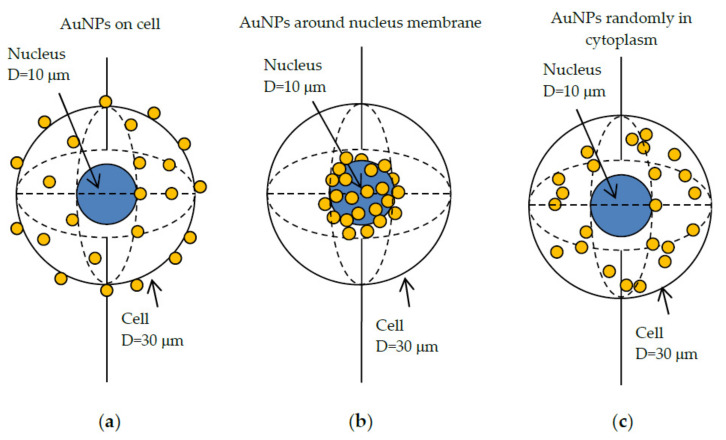
AuNPs distributed in cells: (**a**) AuNPs in the cell surface. (**b**) AuNPs around nuclei membrane. (**c**) AuNPs in cytoplasm.

Two concentric spheres were used to model a simple cell structure. One sphere describes the volume of the complete cell of a diameter of 30 μm, the other one for the nucleus in the cell center with a diameter of 10 μm. Simulations with multiple AuNPs were carried out as shown in Figure 4: AuNPs were distributed (a) in the cell surface, (b) around the surface of nucleus and (c) randomly in cytoplasm [25]. Five sizes of AuNPs in diameters of 100 nm, 74 nm, 50 nm, 14 nm, and 2 nm were used in the simulation. The X-ray spectra applied were 60 kVp, 80 kVp, 100 kVp, 150 kVp, and 200 kVp, covering the energy range of the diagnostic and therapeutic clinical used X-rays [82]. These parallel X-ray beams were sampled from a circular planar source (diameter of 40 µm) along the *z*-axis, which was aligned to the center of the cell. The X-ray beam source is situated at a distance of 50 µm from the cell center.

PARTRAC code [83] was used to simulate the enhancement effects, which can be divided into two parts: one for the physical quantities obtained directly from the track structure, such as primary energy, interaction position, and deposition energy etc.; the other for the biological quantities, which are derived from the superposition of the track structure and the invoked detailed nucleus model. In simulations, the cutoff energy was set as 50 eV for photons and 100 eV and 10 eV for electrons in gold and liquid water, respectively. The radial distribution of average energy deposition within the whole cell volume was restored. The spherical cell model was divided into 15,000 shells. Each shell was 1 nm thick. Hence, 1 × 10^8^ primary photons were transported for the simulation of the physical part. In the biological simulation part, the radiation-induced DNA strand breaks, including single strand break (SSB) and double strand break (DSB), were simulated for the three distributions of multiple AuNPs irradiated by the X-ray beams. The number of primary photons in the biological part was 2 × 10^9^. To quantify the enhancement effect, an additional set of simulations using the identical condition, but without AuNPs was performed. The enhancement factor (EF) was therefore calculated as the ratio of a specific quantity obtained with presence and absence of AuNPs [25].

#### 2.4.4. Multi-Scale Method for Dose, Chemical Radical Generation and DNA Strand Breaks for Multiple AuNPs in a Xenograft Tumor

**Figure 5:**
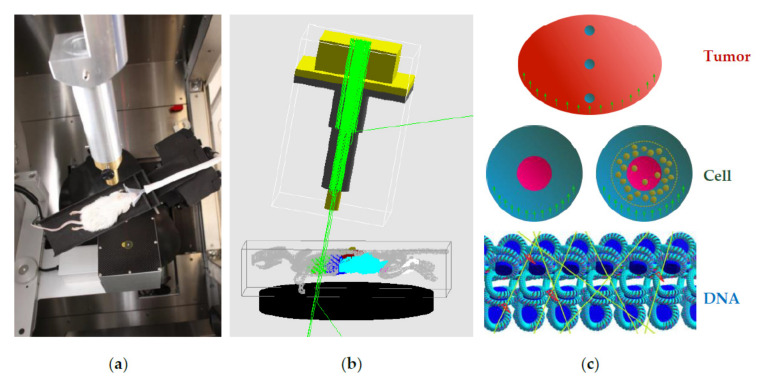
Multi-scale Monte Carlo simulation for enhancement effects with AuNPs distributed in a xenograft tumor model irradiated by X-rays [89]. (**a**) Small Animal Radiation Research Platform (SARRP) with mouse, (**b**) Simulation setup of the SARRP with mouse voxel phantom and (**c**) Computational models of tumor, cell and DNA implemented in TOPAS-nBio.

A new multi-scale methodology for the calculation of DNA damage induced by ionized radiation using TOPAS-nBio was developed [84]. TOPAS-nBio is an extension to the Monte Carlo toolkit TOPAS [85] with a focus on radiobiology. We employed the chemistry interface of TOPAS-nBio, which builds upon the Geant4-DNA radiolysis models [86,87]. We aimed to combine existing optimized simulation parameters with a detailed DNA geometry, while introducing our multi-scale method for a more accurate DNA damage calculation in a realistic experimental setup [31,88]. The model included three steps and each step was connected via phase spaces, which are files storing all particles passing through a chosen surface with all information which is necessary for continuing the simulation (Figure 5). The information stored was particle type, energy, location, and momentum. The following parameters were used in the simulation: cut-off energy: 15 eV, step max: default as 10^6^, the de-excitation process: fluorescence and Auger process turn on, time step and maximum time for chemical stage: 0.5 ps and 999,999 ps. Furthermore, chemical radicals encountering AuNPs are instantly eliminated, meaning that they are unable to produce further DNA damage. There is a considerable discrepancy between the simulations and reality, where interactions between chemical radicals and gold atoms persist and can cause reactions [89].

The simulations were started with a voxel model of a 21 g mouse placed in a box filled with air [90]. To simulate a mammary gland tumor, we defined a small ellipsoid shape close to one of the hind legs as the tumor and replaced all normal tissue in this area by tumor tissue. All simulations were performed twice, with one of two types of kVp photon beams (100 kVp or 200 kVp), which were focused directly to the tumor. The photon spectra were based on the photon source of the Small Animal Radiation Research Platform (SARRP) [91]. Particles entering the tumor were stored in a phase space file. Since a geometry including a whole mouse model is too large for simulation of a realistic radiation dose, we repeated the particles 750 times in the phase space. To increase variance, these duplicated particles were placed on a randomly selected position on the tumor surface, with the momentum rotated accordingly.

The source for the tumor simulations was the phase space from the first step. An ellipsoidal liquid water phantom was defined as the tumor and three spheres representing cells with a diameter of 100 µm were defined at different depths with respect to the initial source. Particles entering these spheres were stored in three respective phase space files. Particles in these files were again duplicated 100 times and each duplicate was rotated randomly.

Cell simulations were performed separately for each of the three depths, each with and without nanoparticles. We used hybrid nanoparticles, made up of a Fe_2_O_3_ core (diameter: 2 nm) surrounded by a gold coating (thickness: 1 nm). The nanoparticles were placed on random positions with up to 100 nm distance to the cell nucleus, which included a previously published detailed DNA model [88,92]. Different Geant4 physics models were used in these simulations. Inside the nanoparticles, Livermore physics were used, whereas detailed Geant4-DNA physics including chemistry module were used in the rest of the cell, which was approximated by water. When at least 17.5 eV of deposited energy caused by physical interactions was scored in a single DNA backbone, it was counted as a direct strand break [93]. If a hydroxyl radical reached or was produced in the DNA backbone, it produced an indirect strand break with a probability of 40% [94,95]. Two strand breaks occurring in a proximity of 10 or fewer base pairs on alternate DNA strands were counted as a double strand break. All other strand breaks were counted as single strand breaks. We also scored the number of produced chemical radicals [89].

### 2.5. Mathematical Modeling of Enhanced Radiobiological Effect

Ionizing radiations excite and ionize water molecules during their path through tissues. Water is the most abundant component of living beings, so it is of paramount importance when studying the radiobiological problem. The primary radiation produces secondary electrons that can interact with DNA directly, inducing single, double, or clustered strand breaks. The electrons are the dominant source for ionizing and exciting molecules in the medium and for the induction of direct DNA damages, independent of the primary particle, from photons to light ions [96]. Low electrons deposit their energy in very small regions and thereby increase the likelihood of more complex DNA damage. This is the reason why Auger electrons are biologically effective and must be involved in in vivo studies with AuNPs. This effect is termed direct physical interaction causing DNA damage.

Once the water molecule has been ionized or excited, it can decay through dissociative channels, producing reactive chemical species, with the OH* radical being biologically most important [97]. This radical can attack DNA by hydrogen abstraction, thereby inducing damage. In addition, secondary electrons can be thermalized and can cause DNA strand breaks via dissociative attachment. This effect is termed indirect chemical interaction causing DNA damage. The enhancement effect around AuNPs leads to both direct and indirect DNA damage [47,98,99,100,101,102,103].

In addition to DNA being the primary sensitive target in a cell, mitochondria are regarded as secondary targets for the AuNP-mediated enhancement effect [104,105] since changes in the mitochondria membrane polarization and oxidation can trigger tumor cell apoptosis. The low energy electron deposited and induced chemical radicals that can result in a loss of cell or nuclear membrane integrity further lead to cell death [30].

There are several proposals to model the biological response based on the direct and indirect damage of DNA and other targets, such as the local effect model (LEM) [106]. This was used to predict the cellular effect induced by AuNP-enhanced effects [107,108,109]. In this study, a mathematical model to estimate the cell survival by using the initial DNA damage data gained by MC simulation has been proposed.

In the 1970s, according to the theory of dual radiation action, both Kellerer and Rossi [110] and Chadwick and Leenhouts [111] proposed the linear-quadratic (LQ) model for the calculation of the cell survival curve. Chadwick and Leenhouts named the LQ model the DSB model, as it linked the cell death with the DSB yield directly. The DSB model is described as

(1) $$S=\exp\left[-p\left(\alpha D+\beta D^{2}\right)\right]$$

where the α*D* represents the number of DSBs proportional to the absorbed dose. These DSBs are caused by the energy deposition events happening at two close positions when an ionizing particle passes through a DNA strand; *βD*^2^ represents the number of DSBs proportional to the square of the absorbed dose. These DSBs are formed by two independent ionizing particles passing through the same DNA strand resulting in energy deposition events at two close locations. Therefore, *αD* + *βD*^2^ represents the number of DSBs generated in a cell for an irradiation with dose *D*, and *p* means the average probability for a DSB to induce cell death. Thus, Formula (1) can be converted to

(2) $$S=\exp\left[-p\left(\alpha D+\beta D^{2}\right)\right]=\exp\left[-p\cdot DSB\right]$$

where the absorbed dose *D* is replaced by the number of DSBs generated in a cell receiving a dose *D.*

Moreover, the ionizing radiation would cause DSBs with different complexities in each cell, which might represent different probabilities of cell death. Therefore, based on Formula (2), the input variable DSB is classified according to its complexity. Assuming there are *i* kinds of DSBs with different levels of complexity, the formula of the cell survival curve can be described as

(3) $$S=q\cdot exp\left[-\underset{i}{\sum }p_{i}DSB_{i}\right]$$

where the *DSB_i_* represents the number of DSBs with *i*th complexity generated in a cell for a dose *D*; *p_i_* refers to the average probability of cell death caused by a DSB with *i*th complexity; *q* represents the ability of a cell tolerating DSB damages, i.e., the cell’s ability to repair DSB, which should decrease with the increase of the number of DSBs. Thus, *q* is further defined as:

(4) $$q=q_{0}+k_{1}\cdot DSB+k_{2}\cdot DSB^{2}$$

In this study, all the DSBs are classified into two kinds based on their complexities: the simple *DSB*_0_ means only one strand break point in each complementary strand within a 10 bp DNA fragment, and the other complex DSBs are defined as DSB_*_. Therefore, the final class I and II models for the assessment of cell death in NASIC (nanodosimetry Monte Carlo simulation code) [81] are expressed as:

(5) $$S=\left(q_{0}+k_{1}\cdot DSB+k_{2}\cdot DSB^{2}\right)\cdot \exp\left[-p\cdot DSB\right]$$

(6) $$S=\left(q_{0}+k_{1}\cdot DSB+k_{2}\cdot DSB^{2}\right)\cdot \exp\left[-p_{0}\cdot DSB_{0}-p_{*}\cdot DSB_{*}\right]$$

The values of *q* and *p_i_* can be fitted with the cell survival results measured from radiobiological experiments, as well as the numbers of DSB or *DSB_i_* with different complexity levels derived from the NASIC simulation of the corresponding experimental irradiation. The fitted results are shown in Table 2.

The DSB damage enhancement factor *EF_DSB_* is defined as:

(7) $$EF_{DSB}=\frac{DSB_{G}}{DSB_{NG}}$$

where the *DSB_G_* and *DSB_NG_* represent the average DSB numbers caused by each source particle with and without AuNP respectively. The cell survival fraction enhancement factor *EF_SF_* is

(8) $$EF_{SF}=\frac{S_{NG}}{S_{G}}$$

where the *S_NG_* and *S_G_* represent the cell survival fractions under the same irradiation without and with AuNP, respectively. Based on assumptions of $S=q\cdot \exp\left[-p\cdot DSB\right]$ and $S=q\cdot \exp\left[-p_{0}\cdot DSB_{0}-p_{*}\cdot DSB_{*}\right]$, Formula (8) can be further converted to

(9) $$EF_{SF}=\exp\left[p\cdot DSB_{NG}\cdot \left(EF_{DSB}-1\right)\right]\;\mathrm{or}$$

(10) $$EF_{SF}=\exp\left[\left(p_{0}a_{0}+p_{*}a_{*}\right)\cdot DSB_{NG}\cdot \left(EF_{DSB}-1\right)\right]$$

In Formulas (9) and (10), the definitions of *p*, $p_{0}$ and $p_{*}$ are the same as Formulas (5) and (6); $a_{0}$ and $a_{*}$ are the fractions of *DSB*_0_ and DSB_*_ in all the DSBs respectively, which sum up as 1. NASIC can simulate the numbers of *DSB*_G_, *DSB*_NG_ and $a_{0}$ in the cell nucleus receiving different irradiations with different photon energies, AuNP sizes and distributions. Based on the values of *p*, $p_{0},$ and $p_{*}$ from Table 2, the cell survival fraction enhancement factor *EF_SF_* can be calculated by Formulas (9) and (10).

## 3. Results

The biological data provided by the laboratory at the Klinikum rechts der Isar [57,69] were used for the computational modelling presented. The results of (1) cmHsp70.1 mAb-AuNP conjugation in vitro and in vivo are initially summarized, followed by our results of (2) the pharmacokinetic model for SPIONs in mice and cellular compartmental model for AuNP conjugation; (3) the dose enhancement around a single AuNP and for multiple AuNPs distributed within a cell; (4) the enhanced cell survival fraction for multiple AuNPs in cells.

### 3.1. Visualization of AuNP Conjugates

#### 3.1.1. Bright-Field and TEM Imaging

**Figure 6:**
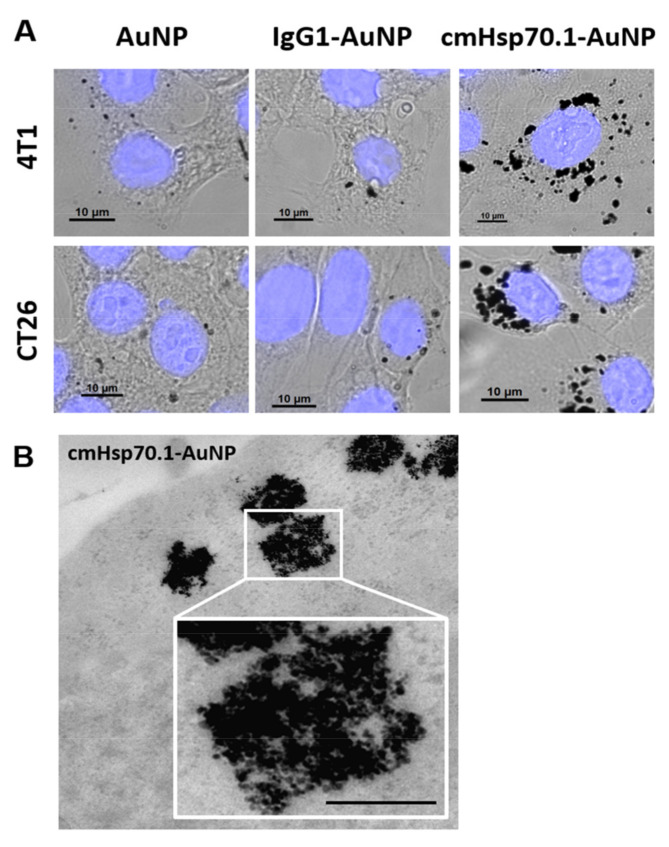
AuNP uptake in cancer cells, in vitro [69]: (**A**) Uptake of AuNPs, IgG1-AuNPs and cmHsp70.1 mAb-AuNPs in cytoplasm and around the nucleus in 4T1 and CT26 tumor cells [69]. TEM images: (**B**) of cmHsp70.1 mAb-AuNPs in 4T1 tumor cells, scale bar, 1 μm. Copyright of *Cancers* (Basel) 2020, 12, 1313; doi:10-3390/cancers12051331 [69].

Three different types of AuNP-conjugates, cmHsp70.1 mAb-AuNPs, and IgG1-AuNPs, and unconjugated AuNPs were incubated with tumor cell lines 4T1 and CT26 for 24 h at 37 °C to simulate the AuNP-uptake [57]. In both tumor cell lines, the uptake of cmHsp70.1 mAb-AuNPs was superior to that of unconjugated or IgG1-control NPs (Figure 6A, [69]). Figure 6B shows the accumulation of cmHsp70.1 mAb-AuNPs in intracellular vesicles of 4T1 cells after 24 h incubation at a higher magnification [69].

#### 3.1.2. Spectral CT Imaging

**Figure 7:**
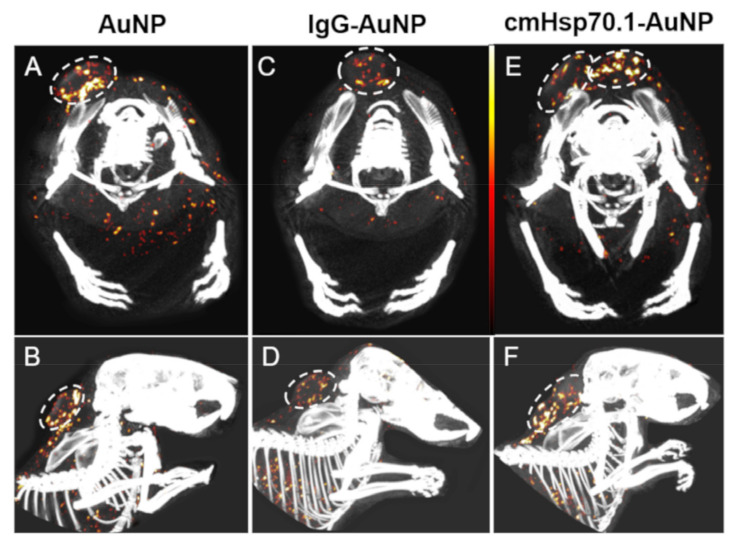
Spectral-CT imaging [69]: upper row for axial view and bottom row for sagittal view. (**A**,**B**) unconjugated AuNP in CT26 tumor; (**C**,**D**) IgG1-AuNP in CT26 tumor and (**E**,**F**) cmHsp70.1 mAb-AuNP in CT26 tumor. Copyright of *Cancers* (Basel) 2020, 12, 1313; doi:10-3390/cancers12051331 [69].

AuNPs were injected into mice bearing subcutaneous CT26 tumors and analyzed by spectral CT imaging, postmortem [69]. The mouse injected with cmHsp70.1 mAb-AuNPs showed the highest concentration of AuNPs (4.4 μg/mm^3^) with a high content at the tumor core (Figure 7E,F). In the mouse injected with control IgG1-AuNPs a lower content of Au (3.3 μg/mm^3^) was detected inside the tumor (Figure 7C,D). In the mouse injected with unconjugated AuNPs, a concentration of 4.3 μg Au/mm^3^ was detected. However, the distribution was largely dispersed over the whole tumor with a main focus at the tumor core (Figure 7A,B) [69]. The spectral-CT images show the distribution of different AuNPs in tumors which were used for MC simulation [89].

### 3.2. Pharmacokinetic Model Developed for SPIONs

**Figure 8:**
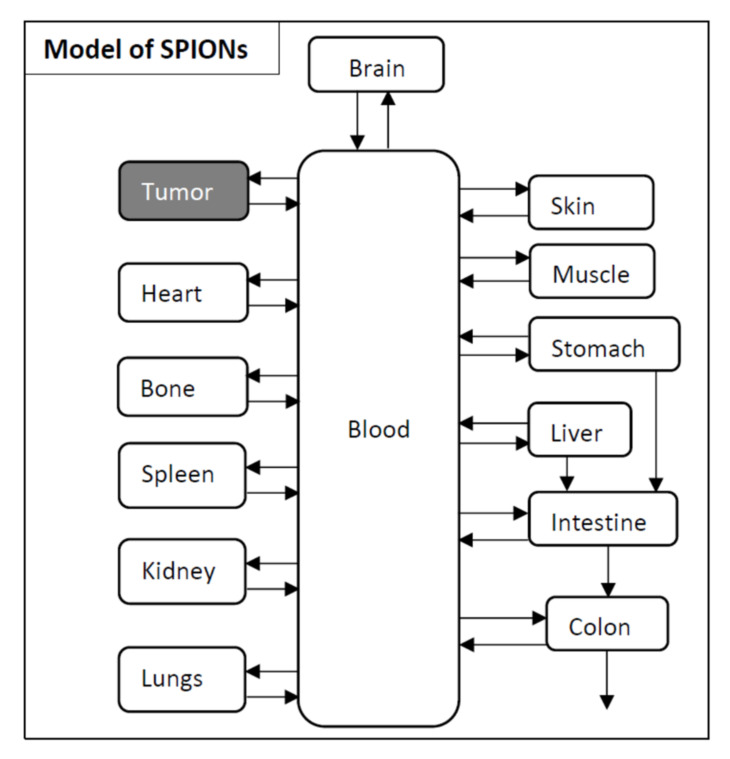
A compartmental model for the pharmacokinetics of biodistribution of SPIONs in mouse.

The pharmacokinetic model based on the PET and SPECT images of SPION nanoparticles coupled with ^89^Zr and ^99m^Tc is illustrated in Figure 8. The transfer rate of the compartmental model is presented in Table 3. The SPIONs are circulating via the blood to the main organs and tissues and return to the blood. This is denoted as a recycling kinetic system. Transfer from stomach to colon through intestinal tract is a non-recycling system. As we do not have any experimental data on urinary excretion from the mice, no urine excretion pathway of SPIONs is included in the model [75].

**Figure 9:**
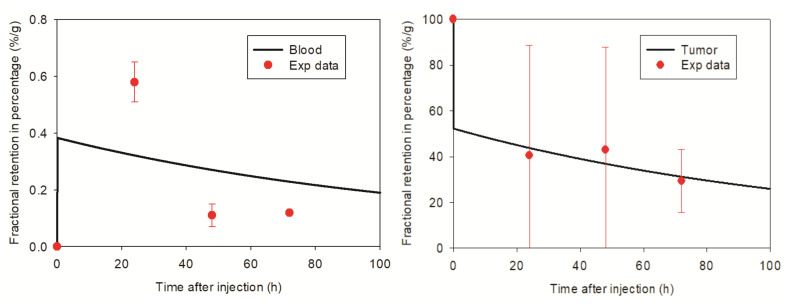

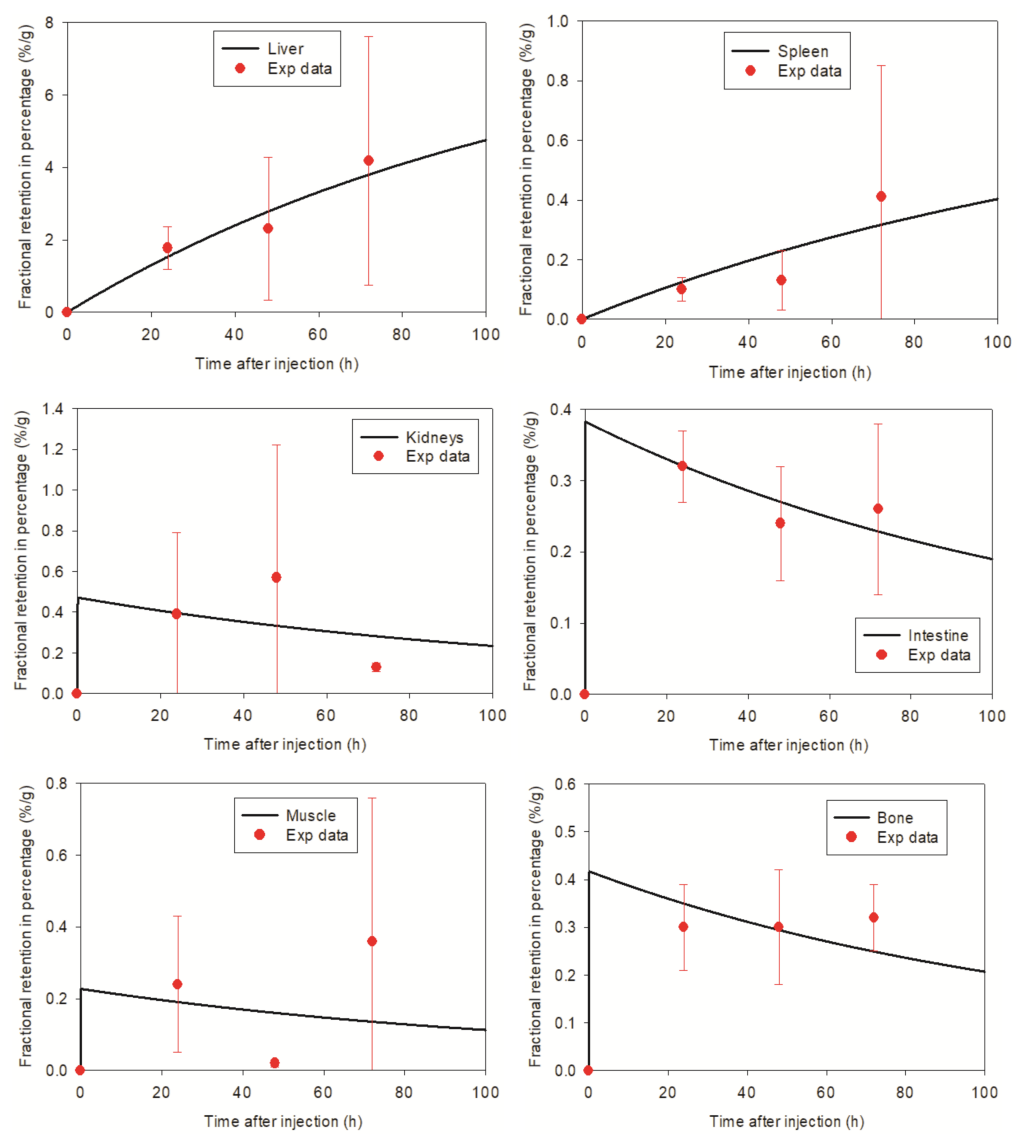
Comparison of model predictions and measured biodistribution data in mice for tumor, blood, liver, spleen, kidneys, muscle, bone and intestine. The red dots and error bars represent the mean values of the measured biodistribution data at a single time point and the standard deviations, respectively. The black curves predict the biokinetic retentions of SPIONs in mice by the pharmacokinetic model and model parameters *k*. shown in Table 3. The y-axes present the percentages of the retained dose per gram after injected amount of SPIONs directly in tumor. “Intestine” denotes the contents of the small intestine in mice. Because of a very low retention fraction, say, <0.1% at each time point, the respective plots for other organs and tissues, such as lungs, skin, heart, colon, etc. are not shown here [75].

The pharmacokinetic model can achieve a time dependent distribution of SPIONs in different organs (Figure 9). This model can be transferred to a model for AuNPs in mice, thereby enabling an irradiation which is adapted to the accumulation of AuNPs in each organ in order to attain the optimal dose enhancement inside the tumor and to reduce the dose in normal organs.

### 3.3. Simulated Dose and Biological Enhancement

#### 3.3.1. Dose Enhancement Ratio for a Single AuNP

**Figure 10:**
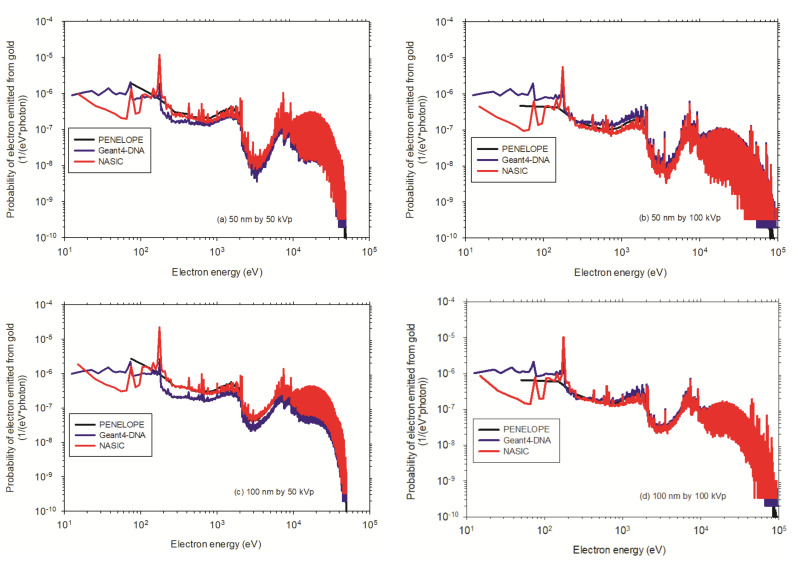
Electron spectra around the AuNP: (**a**) A single AuNP of 50 nm diameter irradiated by 50 kVp X-rays; (**b**) A single AuNP of 50 nm diameter irradiated by 100 kVp X-rays; (**c**) A single AuNP of 100 nm diameter irradiated by 50 kVp X-rays; (**d**) A single AuNP of 100 nm diameter irradiated by 100 kVp X-rays.

Regarding Figure 10, the emission of Auger electrons in gold is dominant in the M-shell and N-shell with a probability of 98.5%. About 33% of Auger electrons have energies of 200–300 eV in the M-shell. In the 200–300 eV energy range of the electron spectra, these Auger electrons primarily contribute to a dose enhancement in the lower nanometer ranges of 10 nm. Electrons of higher energies mostly originate from the interactions of produced X-ray fluorescence with gold inside AuNPs and small parts of Auger electrons with higher energies, of approximately <8 keV. These electrons may contribute to the dose enhancement in the micrometer ranges. The electron energy spectra at low energy, e.g., 100 eV, shows a large variation. For gold, the Livermore physics model in Geant4 was used for the simulation with Geant4-DNA, and the electrons were scored on the surface of the nanoparticle.

**Figure 11:**
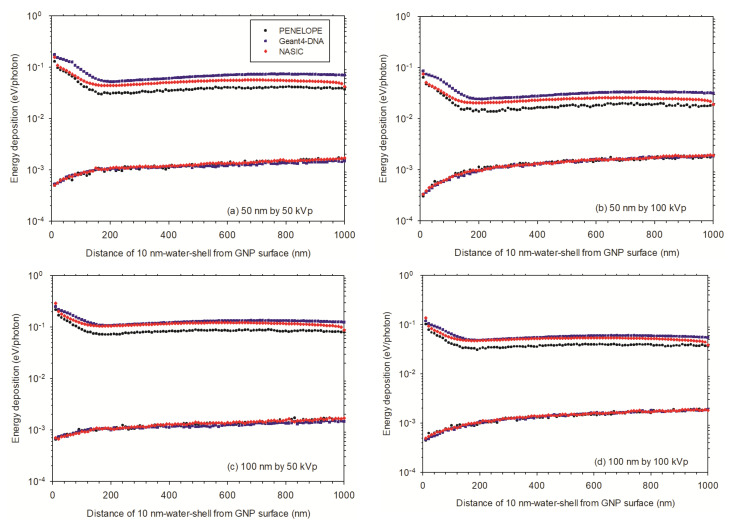
Energy deposition in the 10 nm-thick water shells from surface of the AuNP in the nanometer range: (**a**) A single AuNP of 50 nm diameter irradiated by 50 kVp X-rays; (**b**) A single AuNP of 50 nm diameter irradiated by 100 kVp X-rays; (**c**) A single AuNP of 100 nm diameter irradiated by 50 kVp X-rays; (**d**) A single AuNP of 100 nm diameter irradiated by 100 kVp X-rays. The lower curves are for the energy deposition without AuNP.

In the nanometer ranges, after irradiation with 50 kVp X-rays (Figure 11a,c), the energy deposition (ED) in the water shells around the 100 nm AuNP surface is greater than that of 50 nm AuNPs. With an AuNP present, the ED ranges from 0.4 eV/photon in the first 10 nm-thick water shell to 0.1 eV/photon in the 16th 10 nm-thick water shell for the 100 nm AuNP in comparison to 0.2 to 0.06 eV/photon for the 50 nm AuNP. Without AuNP, the ED in the water shell is very flat. For 100 nm AuNP, the ED starts from 7 × 10^−4^ eV/photon and increases slowly to 2 × 10^−3^ eV/photon, and for 50 nm AuNP, the ED starts from 5 × 10^−4^ eV/photon and increases to 2 × 10^−3^ eV/photon. For the cases of irradiation by 100 kVp X-rays (Figure 11b,d), the ED in the water shell around the 100 nm AuNP surface is also greater than the 50 nm AuNP both in nanometer and in micrometer ranges and with and without AuNP. In the presence of a AuNP, the ED ranges from 0.2 eV/photon in the first 10 nm-thick water shell to 0.05 eV/photon in the 20th 10 nm-thick water shell for the 100 nm AuNP in comparison to 0.1 to 0.04 eV/photon for 50 nm AuNP. Without AuNP, the ED in the water shell is also very flat, for 100 nm AuNP, the ED starts from 4 × 10^−4^ eV/photon and increases slowly to 2 × 10^−3^ eV/photon, and for 50 nm GNP, the ED starts from 3 × 10^−4^ eV/photon and increases to 2 × 10^−3^ eV/photon.

**Figure 12:**
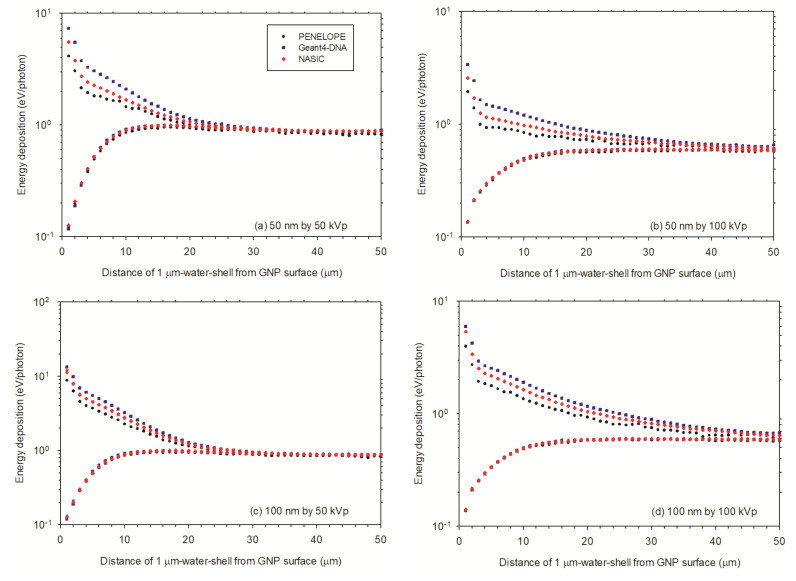
Energy deposition in micrometer range from surface of AuNP: (**a**) A single AuNP of 50 nm diameter irradiated by 50 kVp X-rays; (**b**) A single AuNP of 50 nm diameter irradiated by 100 kVp X-rays; (**c**) A single AuNP of 100 nm diameter irradiated by 50 kVp X-rays; (**d**) A single AuNP of 100 nm diameter irradiated by 100 kVp X-rays. The lower curves are for the energy deposition without AuNP.

In the micrometer range, after irradiation with 50 kVp X-rays (Figure 12a,c), for 100 nm AuNP, the ED starts from 14 eV/photon at the first 1 μm-thick water shell and decreases to 1 eV/photon at 30th 1 μm-thick water shell. It is slightly greater than that for the 50 nm AuNP from 7 to 1 eV/photon at the same water shells. In the absence of AuNP, the ED starts from 0.1 eV/photon at the first and increases to about 1 eV/photon at the 30th water shell for both water nanoparticles. This is understandable, because the ED scored in the 1 μm-thick water shells is less influenced by the 50 nm and 100 nm spheres. For the case irradiated by 100 kVp X-rays (Figure 12b,d), for 100 nm AuNP, the ED starts from 6 eV/photon at the first 1 μm-thick water shell and decreases to 0.7 eV/photon at 40th 1 μm-thick water shell. This is slightly greater than the ED for 50 nm AuNP from 3 to 0.7 eV/photon at the 30th 1 μm-thick water shell. In the absence of AuNP, the ED starts from 0.1 eV/photon at the first 1 μm-thick water shell and increases to about 0.7 eV/photon at 30th water shell for both water nanoparticles. As mentioned before, this is understandable, because the ED scored in the 1 μm-thick water shells is less influenced by the 50 nm and 100 nm spheres.

Taking into account both AuNPs and X-rays (Figure 11 and Figure 12), the ED of 100 nm AuNP irradiated by 50 kVp X-rays reaches the highest value, and 50 nm irradiated by 100 kVp X-rays the smallest values. The ED of 50 nm AuNP irradiated by 50 kVp X-rays and 100 nm AuNP irradiated by 100 kVp X-rays are close each other. For WNP, the ED values are very similar for both AuNP sizes and X-rays, however, the ED of AuNPs irradiated by 50 kVp X-rays are slightly greater than that irradiated by 100 kVp X-rays.

**Figure 13:**
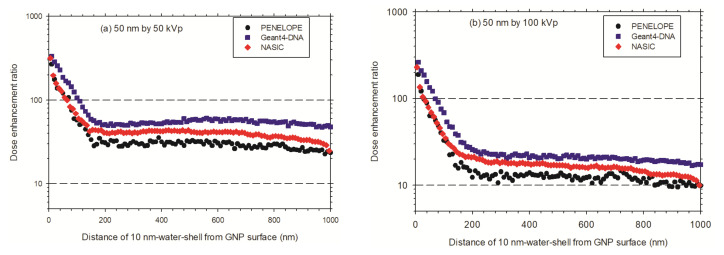

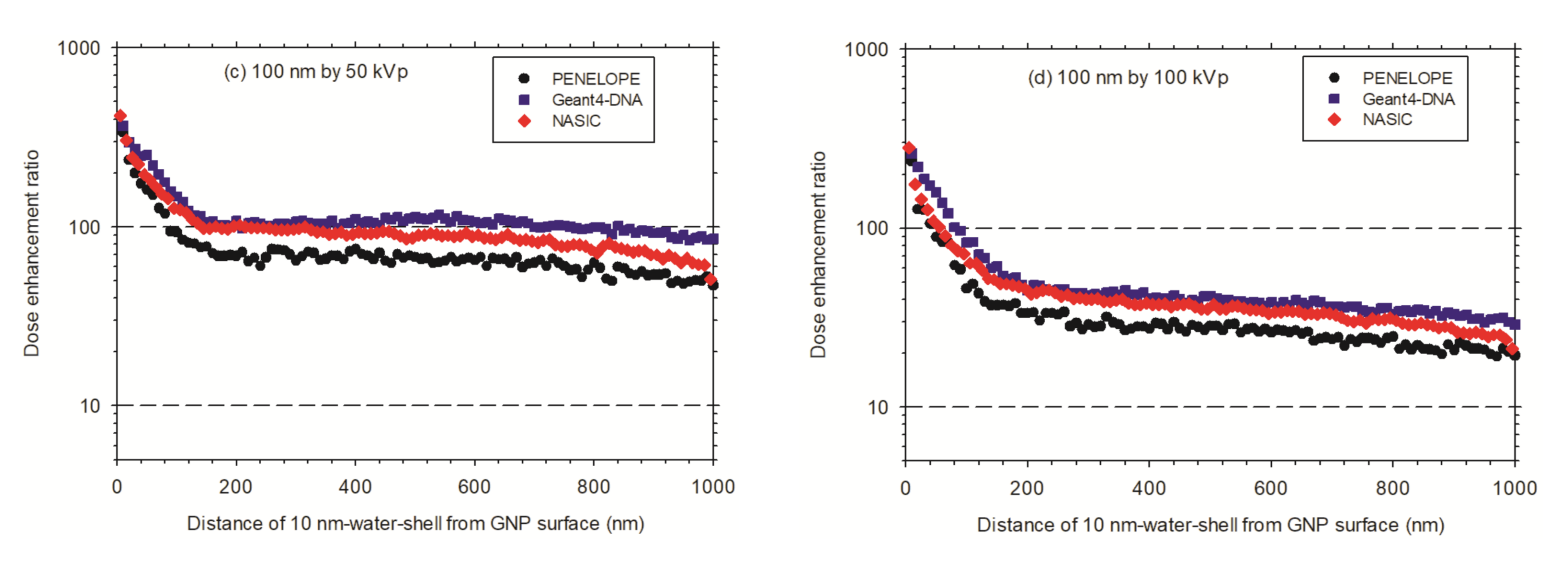
Dose enhancement ratio in the concentric spherical 10 nm-thick water shells from the surface of a single AuNP irradiated by X-rays: (**a**) A 50 nm diameter AuNP irradiated by 50 kVp X-rays; (**b**) A 50 nm diameter AuNP irradiated by 100 kVp X-rays; (**c**) A 100 nm diameter AuNP irradiated by 50 kVp X-rays; (**d**) A 100 nm diameter AuNP irradiated by 100 kVp X-rays.

As shown in Figure 13, in the case of a 50 nm AuNP irradiated by 50 kVp X-rays and 100 kVp X-rays, the DER is about 220 and 180 within the first 10 nm-thick water shell, respectively. In the case of a 100 nm AuNP irradiated by 50 kVp and 100 kVp X-rays, the mean DER within the first 10 nm thick water shell is about 600 and 400, respectively.

**Figure 14:**
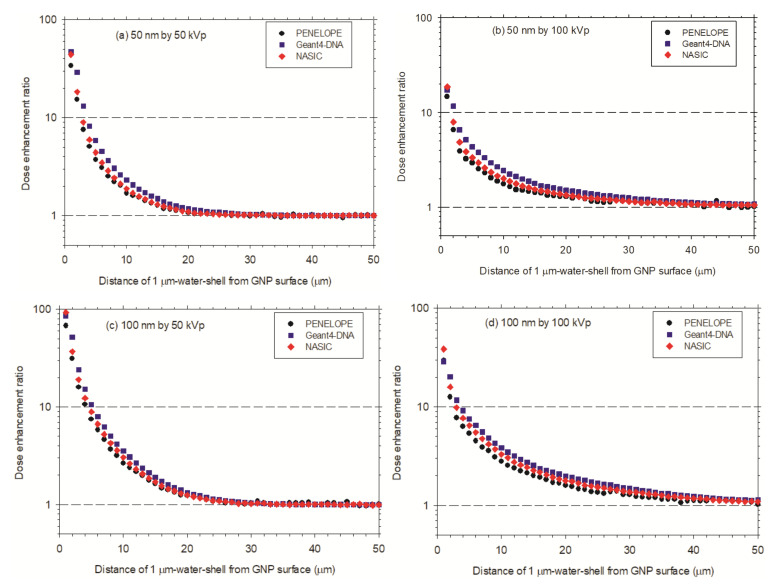
Dose enhancement ratio as a function of the diameter, in the concentric spherical 1 μm-thick water shell distant from the AuNP surface. (**a**) A single AuNP with a diameter of 50 nm irradiated with 50 kVp X-rays; (**b**) A single AuNP with a diameter of 50 nm irradiated with 100 kVp X-rays; (**c**) A single AuNP with a diameter of 100 nm irradiated with 50 kVp X-rays; (**d**) A single AuNP with a diameter of 100 nm irradiated with 100 kVp X-rays.

As shown in Figure 14, in general, in the first 20 μm ranges, the DERs for the AuNP with a diameter of 100 nm are greater than that of the AuNP with a diameter of 50 nm. In distances over 20 μm, the DERs decrease to approximately 1. This indicates that, in the case of AuNPs with diameters of 50 nm and 100 nm after an irradiation with 50 kVp X-rays, the dose enhancement becomes less significant.

There is a difference of ED for a single AuNP irradiated by X-rays sampled from circular and square planar sources (Figure 11 and Figure 12 upper curves). The ED for a circular planar source is slightly greater than that for a square planar source. However, there is no difference of the ED for WNP (Figure 11 and Figure 12 lower curves). Therefore, the DER simulated for a circular planar source is slightly larger than that for a square planar source (Figure 13).

#### 3.3.2. Enhancement Effect of Multiple AuNPs in Cellular Models

**Figure 15:**
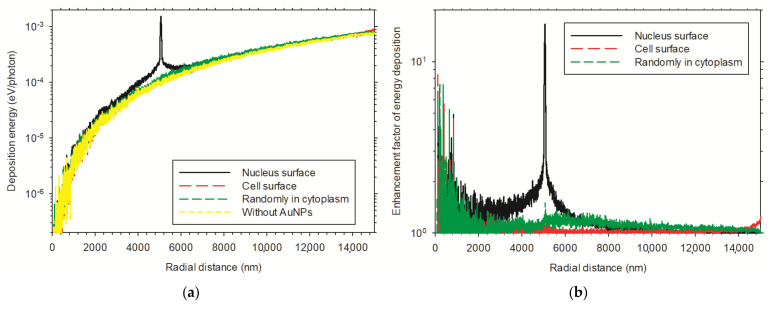
Radial distributions of (**a**) energy deposition and (**b**) enhancement factor within the cell volume for the case of multiple 100 nm diameter AuNPs irradiated by 60 kVp X-rays.

In Figure 15, we present the radial distributions of the average energy deposition and the corresponding enhancement effects for the multiple 100 nm diameter AuNPs distributed in cells and irradiated by 60 kVp X-rays. For the (b) distribution shown in Figure 4, a significant peak of energy depositions can be seen within the few hundred nanometers extension from the nucleus surface shell which was distributed by AuNPs. The curve for the AuNPs randomly distributed in cytoplasm is relatively flat and only slightly above the curve in the case of without AuNPs (Figure 15a). Enhancement effects showed the highest value of 10 for AuNPs distributed around the nucleus surface, with a fluctuant part close to the center (Figure 15b). For AuNPs that are randomly distributed in the cytoplasm, the enhancement factor ranges from 1 to 2, with an average value about 1.1 [25].

**Figure 16:**
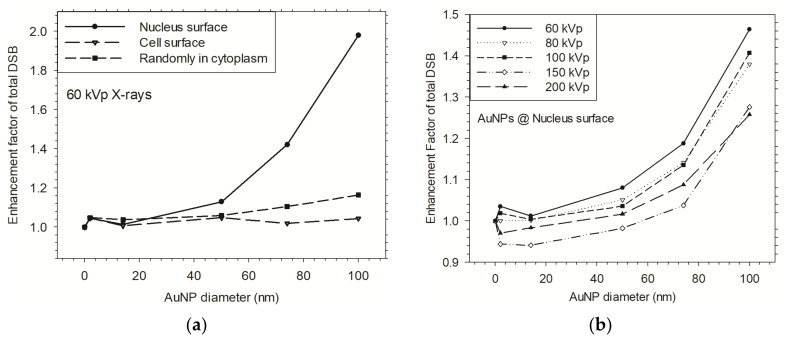
Enhancement factor of induced total DSB as a function of the size of multiple AuNPs in cells. (**a**) Multiple AuNPs under different distributions in cells and irradiated by 60 kVp X-rays. (**b**) Multiple AuNPs were distributed around the nucleus surface and irradiated by various X-rays, from 60 to 200 kVp.

We defined DNA strand breaks induced by direct physical tracks and indirect chemical radicals as a biological enhancement effect endpoint (Figure 16). The enhanced radiosensitization effect is caused by AuNPs which were distributed in the cellular environment and were irradiated by X-rays. There are several other factors which were not taken into account in the simulation that can influence the enhancement effect. However, the presented results imply that the irradiation condition of AuNPs distribution on the nucleus surface by the 60 kVp X-rays may reach the most effective enhancement effect in the various distributions of multiple AuNPs in cells and the x-ray irradiation sources [25].

#### 3.3.3. Enhancement Effect of Multiple AuNPs in Xenograft Tumor Model

**Figure 17:**
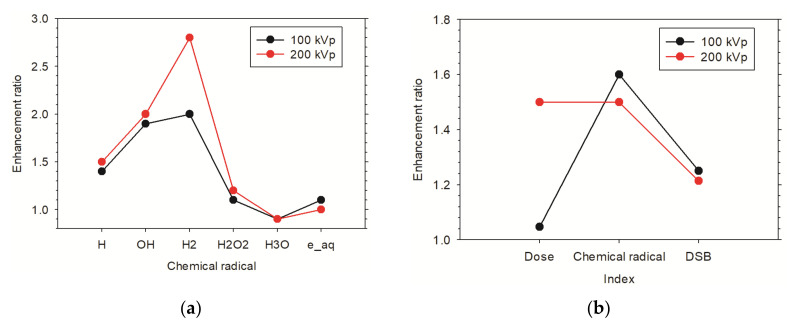
Enhancement ratio of (**a**) dose in to nucleus, produced chemical radicals and total DSB and of (**b**) different chemical radicals produced when a fixed number of AuNPs are distributed around nucleus irradiated by 100 kVp and 200 kVp X-rays from the front direction.

Dose enhancement at cellular levels is small for both X-ray irradiations. DER for 200 kVp is about 1.5 and only 1.1 for 100 kVp. The enhancements ratios of chemical radical and DSB in both X-rays are at a similar level, respectively (Figure 17b). The DER of DSB obtained within TOPAS-nBio is comparable to the results simulated by PARTRAC code [25]. Figure 17a showed the DERs of various chemical radicals produced around AuNPs. Generally, the radicals produced by 200 kVp are much more enhanced than that of 100 kVp X-rays. It is shown that more radicals OH and H_2_ are produced than others [89]. This information is crucial to understand the contribution of chemical enhancement to the biological enhancement.

### 3.4. Modeled Enhanced Radiobiological Effect—Cell Survival Fraction

**Figure 18:**
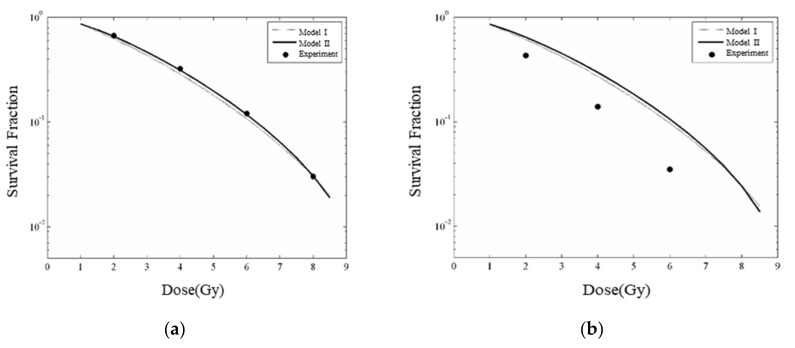
Comparison of calculated cell survival fractions based on the cell death mode I ($S=q\cdot \exp\left[-p\cdot DSB\right]$) and II ($S=q\cdot \exp\left[-p_{0}\cdot DSB_{0}-p_{*}\cdot DSB_{*}\right]$ ) proposed in Section 2.5 with experimental results. The irradiation source is 220 kVp X-rays. (**a**) Without AuNP; (**b**) 6000 AuNPs with a diameter of 50 nm which are randomly distributed in the cytoplasm.

Without AuNP in cells, the calculated cell survival fraction, based on MC simulated DSBs, is in good agreement with the experimental results [41] (Figure 18a). However, as AuNPs are widely distributed in cells, the increase of DSBs in simulations is smaller than shown in experiments (Figure 18b). The main reason for this phenomenon is that the current simulation assumes that all AuNPs were randomly distributed in the cytoplasm, and only 10~20% of AuNPs can enhance the radiation sensitization effect in the nucleus, however, the real distribution of AuNPs in the cell is yet unknown. To realistically assess the radiation sensitization effect of AuNPs in cells, the biological dynamic process and biodistribution of AuNPs in the cellular environment need to be considered in the MC simulation and radiobiological modeling.

## 4. Discussion

The conjugation of AuNPs to cmHps70.1 antibody provides a promising tool to selectively target tumor cells for a potential AuNPs-aided targeted cancer radiotherapy in the future. The in vivo and in vitro microscopic images of the distribution of cmHsp70.1-AuNP-conjugates within cancer cells [57,69] enabled the setup of cellular compartmental models to estimate the kinetic behavior of AuNPs after in vivo application. To overcome the limits of the model to small animals, continuum models for nanoparticle transport may be successfully applied, such as that reported by Wirthl et al. [112]. In that case, larger domains, such as multiphase tumor growth and longer time scales, can be investigated. This information can be used in the MC simulation for quantifying the radiation dose, chemical radicals, and the biological dose enhancement effect. Such dose and chemical enhancement information can be used to assess the experimental radiobiological effects, and response relationships may be established for clinical translation of this high potential radiosensitizer AuNPs in radiotherapy treatment planning.

In traditional therapy, the dose–effect relationship is mainly based on the dose in a macroscopic scale. However, the AuNP-aided radiotherapy depends on many physical characteristics, such as composition, size, shape, coating and concentration, degree of uptake, and subcellular localization within a cell. Therefore, the heterogeneity of dose at nano- and micro-macroscopic levels strongly impacts the dose–effect relationship. Specifically, the DER in different scales shows different results depending on the targets, organs, tissues, cells, and DNA.

The DER for a single AuNP irradiated with a very narrow kVp X-ray beam in the nano- and micrometer ranges showed a greater value, e.g., 500 with the first 10 nm water shell around the surface of the single AuNP. However, this high DER value should be corrected to a lower value because of the narrow X-ray beam which is merely 10 nm greater than the size of a single AuNP. A correction method is proposed for reducing the higher DER to a lower value [113]. However, this correction method underestimated the DER, because only the photon interactions from a broader beam with liquid water were considered. These photons can interact with AuNP and produce further Auger electrons and X-ray fluorescence electrons. Therefore, the corrected DER value irradiated with a narrow beam may be 40–50 for a single AuNP irradiated with a broader X-ray beam. These values are similar to the results with multiple AuNPs and a broader X-ray beam [23,28]. However, these data must be tested and validated by further MC simulations with a broader X-ray beam and the phase-space file method. Furthermore, the new cross sections [95,114] for gold interaction with photons and electrons in the lower energy range should be integrated into the commonly used MC codes, like Geant4-DNA, so that a much more precise energy deposition around the surface of the AuNP may be obtained.

DER at the cellular level is generally low due to the greater scoring volume because energy contributing to the dose enhancement originates from electrons with lower energy. The enhanced energy will be averaged over the total cellular volume. Therefore, in the authors’ opinion, the DER in cellular level is not an appropriate index for the dose–effect relationship, because the cell death and killing represent later effects of intimal damages to DNA and other organelles in cells, which receive energy in the hundred nanometer range. In multi-scale MC simulations of the radiobiological effects, the physicochemical and chemical module has not yet been fully implemented. Therefore, the contributions to enhancement effects from the radiation induced chemical radical damage to DNA and other molecular structures need further investigations. Furthermore, there are other enhancement effects induced by irradiations with X-rays, such as charge transfer [115], enhanced permeability and retention [116], and surface plasmons [117], which can cause further radiation-induced damage. These effects can now be simulated with the newly published Geant4-DNA-Au package [114], which includes a plasmon excitation model implemented into the MC code Geant4-DNA.

Besides the real dose in the macroscopic scale and the sub-tissue dose as well as the microscopic dose, there are many other challenges in accurate treatment planning with AuNPs in radiotherapy, such as the concentration of AuNP in macroscopic scale. One challenge is to ensure the accurate transfer of AuNP-conjugates to the cancerous cell via the circulation system. Radiobiological experiments with different AuNPs-conjugates must be carried out to provide the real enhancement effects under different physical, chemical, and biological conditions. The enhanced radiation dose adopted in the present study is only a very first step in the simulation of all radiobiological effects. Only solid experimental radiological results and real time imaging controls of the distribution of AuNPs in the tumor can provide enough data to translate targeted AuNP therapies into clinical practice.

In conclusion, further experimental research activities are necessary to accelerate the translation of AuNPs in targeted clinical cancer radiotherapy.

1. Dose enhancement of a single AuNP in liquid water irradiated by a broad beam. These values might be used for the dose enhancement of multiple AuNPs in liquid water.
2. Dose enhancement of a single AuNP coated with different tumor-specific antibodies, such as cmHsp70.1 in combination with an irradiation using a broad X-ray beam to assess the reduction of the dose enhancement [118,119].
3. Evaluation of the radiation-induced chemical radicals contributing to the enhancement effects of multiple AuNPs to the biological effects with advanced chemical module-implemented MC simulations.
4. The cellular or molecular compartmental model for the heterogeneous uptake and distribution of AuNP-conjugates on the cell membrane and in the cytoplasm.
5. Radiobiological experimental setup and defined endpoint results with injections of a controlled amounts of AuNPs with known shape composition and surface modifications, in vivo.
6. Further advanced optical imaging technique for initial dynamic process of AuNPs in cells prior to and after irradiation.

## 5. Conclusions

AuNP conjugated to tumor-targeting antibodies or other molecules can improve radiotherapy by reducing the total radiation dose and thereby minimizing radiotoxicity to neighboring organs at risk. This paper summarizes the progress and main results of a joint gold nanoparticle-aided radiotherapy program.

Based on existing datasets obtained by our group, a pharmacokinetic model was developed that describes the distribution of SPIONs in mice and it demonstrated the feasibility of the model for AuNPs in mice. A Monte Carlo radiation transport simulation was performed on a single AuNP and multiple AuNPs in multiple scales, i.e., in tumors, cells and at the molecular level, to provide the enhanced radiation dose and chemical radicals in close proximity to AuNPs. Furthermore, a mathematical model was established to predict the biological responses to doses enhanced by AuNPs irradiated with X-rays.

Images showed the distribution of AuNPs in the cancer and its microenvironment observing an accumulation of cmHsp70.1-AuNPs inside the tumor core [69]. Further, time-dependent cellular imaging of AuNPs is needed to develop a pharmacokinetic model for predicting the biodistribution of AuNP-conjugates at the cellular level. Simulated radiation dose enhancement for a single AuNP demonstrated enhanced dose effects. However, the simulated chemical radicals and DNA strand breaks for multiple AuNPs and multiple target scales showed only a moderate enhancement effect. The modeling of radiobiological enhanced effects of AuNPs can predict the cell survival and may guide ongoing radiobiological experiments with AuNP-conjugates.

We could predict the biodistribution of nanoparticles in small animals using a pharmacokinetic model. Simulated enhancement effects derived from the physical dose and chemical radicals showed that conjugated AuNPs have the potential to be translated in targeted radiotherapy in the future. However, many physical, chemical, and biological parameters still need further investigation.

## 6. Patents

Stefan Stangl and Gabriele Multhoff for the patent: Peptide-based compounds and their uses for tumor imaging and targeting. EP14172984 Klinikum Rechts der Isar, Technische Universität München. 2014.

## Figures and Tables

**Table 1:** Main parameters utilized to generate the X-ray spectra.

Parameter	Unit	Values
Peak voltage	kVp	50, 60, 80, 100, 150 and 200
Energy bin	keV	0.5
Angle theta	degree	20
Air thickness	mm	470
Beryllium thickness	mm	0.8
Aluminum thickness	mm	3.9
Nf		0.68
P		0.33

**Table 2:** The fitted results of cell survival model parameters in NASIC.

DSB Class	*Q*			*p*		*R* ^2^
	$q_{0}$	*k*_1_ (10^−3^)	*k*_2_ (10^−5^)	Notation	Value (10^−3^)	
I	1.090	5.917	−1.879	*p*	9.944	0.9893
II	1.037	7.275	−2.296	$p_{0}$	3.088	0.9987
				$p_{*}$	30.68	

**Table 3:** Systemic transfer rates (*k*) from and to blood and the transfer rates in the alimentary tract in mouse [75].

Systemic Circulation	Transfer Rate to Blood (h^−1^)	Clearance Rate from Blood (h^−1^)
Tumor	327.4	40.06
Liver	0.1159	5.067 × 10^−4^
Spleen	9.78 × 10^−4^	1.409 × 10^−5^
Kidneys	3.804	15.66
Colon	0.7151	689.6
Stomach	7.411	896.0
Lungs	1.903	148.5
Small intestine	68.93	59.41
Muscle	194.2	62.39
Bone	62.6	38.58
Skin	9.38	27.58
Brain	5.439	795.6
Heart	2.416	490.5
**Alimentary tract**		
Donor	Receptor	Transfer rate (h^−1^)
Liver	Small intestine	4.947 × 10^−4^
Stomach	Small intestine	64.71
Small intestine	Colon	3.447
Colon	Excretion	4.244 × 10^−3^

## Data Availability

The data presented in this study are available in this article.
